# Progression of Mouse Skin Carcinogenesis Is Associated with Increased Erα Levels and Is Repressed by a Dominant Negative Form of Erα

**DOI:** 10.1371/journal.pone.0041957

**Published:** 2012-08-03

**Authors:** Stella Logotheti, Dimitra Papaevangeliou, Ioannis Michalopoulos, Maria Sideridou, Katerina Tsimaratou, Ioannis Christodoulou, Katerina Pyrillou, Vassilis Gorgoulis, Spiros Vlahopoulos, Vassilis Zoumpourlis

**Affiliations:** 1 Biomedical Applications Unit, Institute of Biology, Medicinal Chemistry and Biotechnology, National Hellenic Research Foundation, Athens, Greece; 2 Cryobiology of Stem Cells, Centre of Immunology & Transplantation, Biomedical Research Foundation, Academy of Athens, Athens, Greece; 3 Molecular Carcinogenesis Group, Department of Histology and Embryology, Medical School, University of Athens, Athens, Greece; Emory University, United States of America

## Abstract

Estrogen receptors (ER), namely ERα and ERβ, are hormone-activated transcription factors with an important role in carcinogenesis. In the present study, we aimed at elucidating the implication of ERα in skin cancer, using chemically-induced mouse skin tumours, as well as cell lines representing distinct stages of mouse skin oncogenesis. First, using immunohistochemical staining we showed that ERα is markedly increased in aggressive mouse skin tumours *in vivo* as compared to the papilloma tumours, whereas ERβ levels are low and become even lower in the aggressive spindle tumours of carcinogen-treated mice. Then, using the multistage mouse skin carcinogenesis model, we showed that ERα gradually increases during promotion and progression stages of mouse skin carcinogenesis, peaking at the most aggressive stage, whereas ERβ levels only slightly change throughout skin carcinogenesis. Stable transfection of the aggressive, spindle CarB cells with a dominant negative form of ERα (dnERα) resulted in reduced ERα levels and reduced binding to estrogen responsive elements (ERE)-containing sequences. We characterized two highly conserved EREs on the mouse ERα promoter through which dnERα decreased endogenous ERα levels. The dnERα-transfected CarB cells presented altered protein levels of cytoskeletal and cell adhesion molecules, slower growth rate and impaired anchorage-independent growth *in vitro*, whereas they gave smaller tumours with extended latency period of tumour onset *in vivo*. Our findings suggest an implication of ERα in the aggressiveness of spindle mouse skin cancer cells, possibly through regulation of genes affecting cell shape and adhesion, and they also provide hints for the effective targeting of spindle cancer cells by dnERα.

## Introduction

Skin cancer, both non-melanoma and melanoma, presents an alertingly increasing incidence rate worldwide, followed by substantial mordibity and raise of treatment cost. Risk factors for non-melanoma skin cancer include excessive exposure to sunlight or ultraviolet light, fair complexion and occupational exposure to certain chemicals [1], as well as organ transplantation [2]. Although mortality due to primary non-melanoma skin cancer is infrequent, prognosis is rather poor when metastasis occurs. The five-year survival rates in case of metastatic non-melanoma skin cancer are low, as a result of limited effectiveness of aggressive treatment of metastatic carcinomas with radiation and/or chemotherapy agents such as methotrexate, fluorouracil and bleomycin [1].

An effective approach for investigating skin cancer is the mouse skin carcinogenesis model developed previously, following a protocol of chemical treatment of mouse epidermis with 7,12-dimethylbenz[a]anthracene (DMBA) and 12-O-tetradecanoylphorbol-13-acetate (TPA). This model consists of a series of cell lines isolated from mouse tumours following a two-staged chemical carcinogenesis protocol, which represent distinct stages of the full range of mouse skin cancer development. It includes, but is not limited to, an immortalized, non-tumourigenic keratinocyte cell line (C5N), a benign papilloma cell line (P1), a squamous carcinoma cell line (B9), which gives rise to well differentiated tumours upon injection into nude mice, and finally highly anaplastic, invasive spindle cell lines (A5, CarB) that exhibit aggressive metastatic tumour growth *in vivo*. Although an artificial system, it is ideal for analysis of the events that lead to the transition through the stages of initiation to promotion and finally, to progression of carcinogenesis [3].

Estrogen receptors (ERs) are transcription factors that regulate a variety of genes both directly (ERE-containing genes, i.e. Estrogen Responsive Element-containing genes) and indirectly (non-ERE containing genes, i.e. non-Estrogen Responsive Element-containing genes) and are implicated in oncogenic and apoptotic events in a diverse range of estrogen-responsive target tissues [4], [5]. There are two types of estrogen receptors, ERα and ERβ, which are encoded by distinct genes, present differential tissue-specific expression patterns [4] and form homo- as well as hetero-dimers, when co-expressed in the same tissues. Since they share a 96% amino acid identity in their DNA-binding domains (DBD), they can bind to target sequences with similar affinities. The lower (53%) homology in their ligand domains accounts for the differences in their responses to various ligands (e.g. tamoxifen, raloxifen and phytoestrogens), whereas their even less conserved N-terminal transactivation domains enable interactions with different proteins in the transcription complexes, thereby differentiating their effects on target genes [6]. It has been suggested that a ‘yin-yang’ relationship exists between ERα and ERβ, since ERα induces proliferation whereas ERβ induces apoptosis in the normal murine mammary cell line HC11 [7]. This antagonistic relationship is further supported by the fact that ERα and ERβ exert opposing activities on activated protein-1 (AP-1) sites [8]. For instance, the AP-1-driven promoter of cyclin D1 is activated by ERα, but repressed by ERβ [9]. This evidence has led to the notion that it is the ERα/ERβ ratio rather than the individual receptor levels that is associated with carcinogenetic processes in tissues where both ERα and ERβ are expressed [10], [11]. Indeed, the ERα/ERβ ratio has been found to be elevated in breast cancer [12], as well as in uterine leiomyomas [13].

A recent report has highlighted a significant role of ERs in nonmelanoma skin cancer, focusing particularly at the promotion stage of chemically induced mouse skin oncogenesis. More specifically, ovariectomized female mice, susceptible to papilloma and squamous cell carcinoma (Car-S mice), presented an increase in the papilloma incidence upon treatment with chemical carcinogens in comparison with the intact control mice. Furthermore, the ERα/ERβ ratio increased in papillomas from DMBA/TPA-treated, ovariectomized Car-S mice compared to DMBA/TPA-induced papillomas from, non-ovariectomized control animals, as a combined result of both elevated ERα levels and reduced ERβ levels. This increase subsequently led to the upregulation of the typical ER target, cyclin D1, and of the proliferation marker Ki67 in these animals [14].

Following this work and exploiting the well-established mouse skin carcinogenesis model, we aimed to shed more light on the role of ERs in the full spectrum of skin carcinogenesis, especially in the progression stages, both *in vitro* and *in vivo*. We first estimated the alteration in the expression of ERα and ERβ in spindle skin tumours from DMBA/TPA-treated mice compared to the papilloma tumours. Then, we investigated ER levels and ER binding activity in regards to skin cancer initiation, promotion and progression stages (represented by C5N, P1, B9, A5 and CarB cell lines) and we emphasized on the *in vitro* and *in vivo* effects of ERα-specific targeting in the aggressive characteristics of CarB by S554fs, a dominant negative mutant of ERα (dnERα). We also clarified a new mechanism through which S554fs can exert its inhibitory activities.

## Results

### Increased Levels of ERα and Decreased Levels of ERβ are Observed in Advanced Stages of Mouse Skin Carcinogenesis

**Figure 1:**
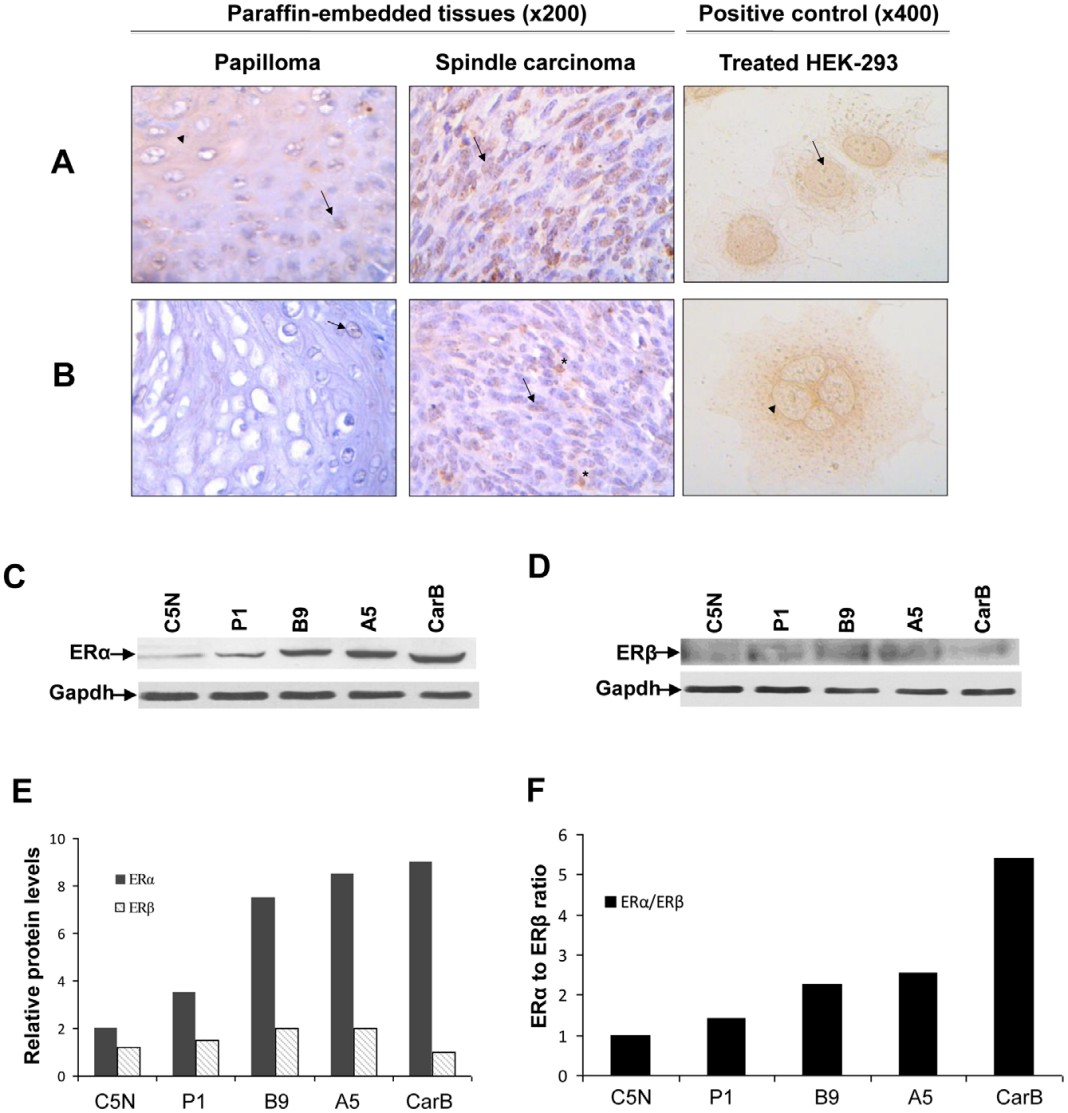
Changes in *ERα* and *ERβ* expression in skin carcinogenesis *in vitro* and *in vivo.* (A) Immunohistochemical expression of ERα is enhanced in serial section of a spindle cell carcinoma in comparison with skin papilloma derived from chemical induction of carcinogenesis in athymic mice. (B) Immunohistochemical staining of ERβ is faint in spindle cell carcinomas in comparison with skin papillomas. Nuclear staining of the positive control ERα-HEK293 cells and cytoplasmic staining of the ERβ-HEK293 cells, which are used as positive controls validate the efficiency of anti-ERα and anti-ERβ, correspondingly, in this experimental setup. *Symbols:* Block arrows indicate positively stained nuclei, while arrowheads indicate positively stained cytoplasm. The asterisks show macrophages. (C) Western blot with anti-ERα in total extracts of C5N, P1, B9, A5 and CarB cell lines reveals that ERα is enhanced during transition from the immortalized C5N cell lines to the most aggressive CarB cell lines. (D) Western blot with anti-ERβ reveals slight changes of ERβ during transition from the immortalized C5N cell lines to the most aggressive CarB cell lines. GAPDH protein expression was used as a loading control. (E) Quantification of ERα and ERβ levels in C5N, P1, B9, A5 and CarB cell lines (F) ERα/ERβ ratio is gradually increased during transition from the immortalized C5N through most aggressive CarB cell line.

Initially, the status of ERα and ERβ in early (papillomas) and advanced (spindle tumours) stages of mouse skin carcinogenesis *in vivo* was studied. Immunohistochemical expression of ERα was assessed in chemically induced skin tumours from eight mice. In the papillomas examined, only a faint nuclear staining for ERα was observed, while spindle cell carcinomas exhibited a strong nuclear staining for ERα (Fig. 1A). In contrast, ERβ levels were very low in papillomas and they were almost undetectable in spindle cell carcinomas (Fig. 1B). To ensure that the antibodies used on the paraffin sections are working properly, stable transfected ERα-expressing HEK-293 cells and ERβ-expressing HEK-293 cells [15] were used as positive controls for staining with anti-ERα and anti-ERβ antibodies, correspondingly.

### The ERα/ERβ Ratio Increased During Promotion and Progression Stages of Mouse Skin Carcinogenesis

The levels of both ERα and ERβ in the multistage mouse skin carcinogenesis cell lines C5N, P1, B9, A5 and CarB were then estimated. We found ERα to be significantly elevated through transition from the immortalized C5N cell line (initiation stage) to the papilloma (promotion stage), squamous and spindle cell lines (progression stages), overall presenting the highest levels of expression in the CarB cell line. Notably, the most profound increase in ERα levels was observed during transition from the benign P1 cell line to the tumourigenic B9 cell line (Fig. 1C, 1D). On the other hand, the protein levels of ERβ in P1, B9 and A5 cell lines only slightly changed in comparison with C5N, with the lowest levels being those in the CarB cell line (Fig. 1D, 1E). The corresponding ERα/ERβ ratio gradually increased from the C5N through the CarB cell line, being the highest in the most aggressive CarB cells (Fig. 1F).

### dnERα Reduces ERα Levels in the Spindle CarB Cell Line

**Figure 2:**
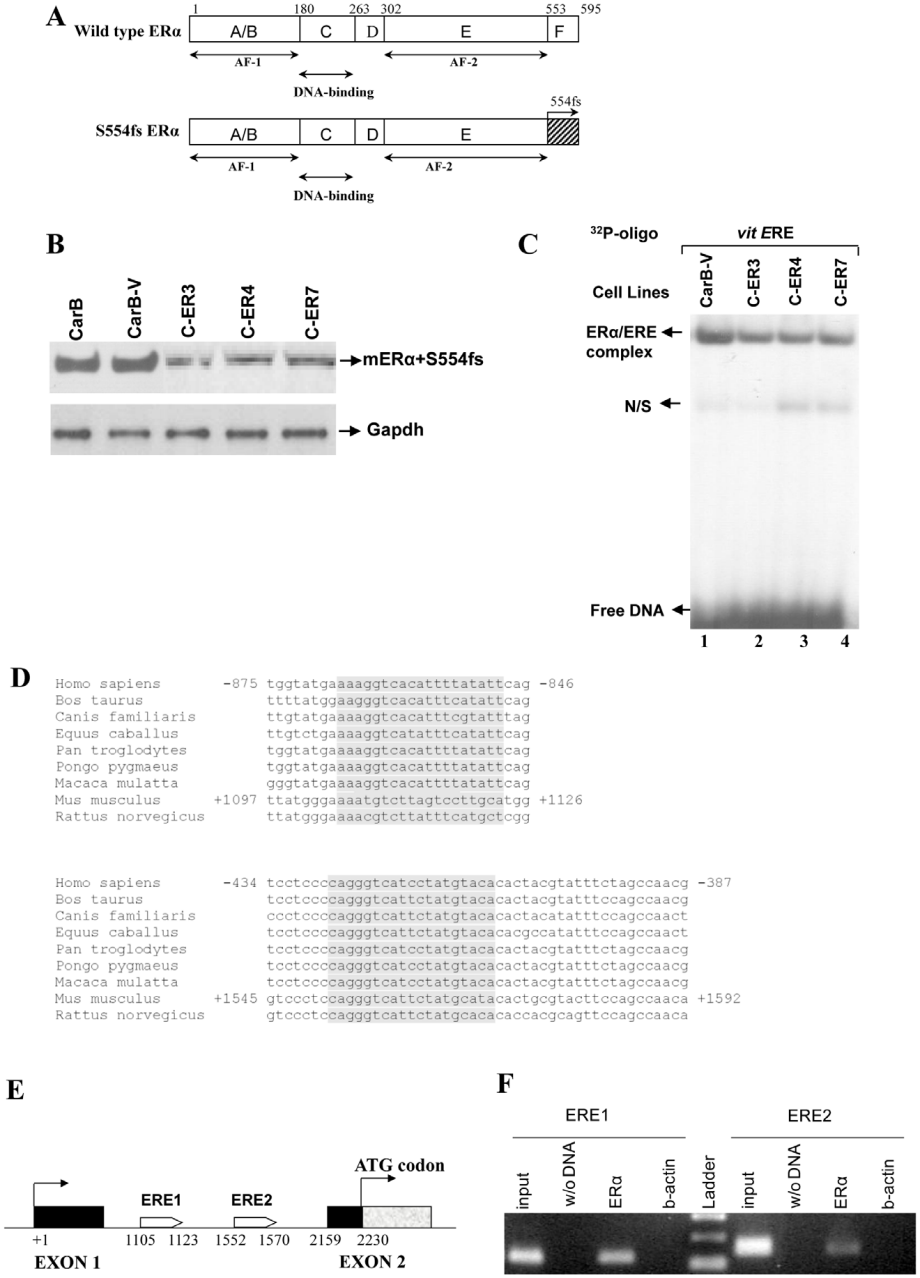
Effect of the dominant negative S554fs-ERα on ERα levels of CarB cells. (A) Wild type and mutant form of ERα. The *S554fs-*ERα was generated by random chemical mutagenesis of the ERα hormone binding domain (19). (B) Western blot analysis in equally-loaded protein extracts from parental, control vector-transfected (CarB-V) and dnERα-transfected cells (C-ER3, C-ER4 and C-ER7) with an antibody that recognizes mouse and human ERα (wild type and mutant). The levels of the total ERα protein (endogenous wild type plus exogenous mutant ERα) in all three clones compared to the baseline ERα levels in CarB and CarB-V cells are reduced. (C) Electrophoretic mobility shift assay for ERα demonstrated lower binding activity of ERα dimers to the estrogen response element of the vitellogenin promoter, *vit*ERE, in the stable transfectant cell lines (lanes 2,3 and 4) compared to the control CarB-V cells (lane1); N/S: non-specific binding. (D) The mouse *ERα* gene contains 2 putative estrogen responsive elements (grey-highlighted), highly conserved among several animal species. (E) Schematic representation of the location of the putative EREs on the mouse *ERα* gene. Two EREs (white blocks) are contained within intron 1–2 of *ERα*. The black shades indicate untranslated regions of exons 1 and 2, whereas the grey shade indicates translated region of exon 2. The ATG codon is identified at +2230. (F) ChIP assay with DNA from CarB cells, using antibody against ERα and PCR primer pairs that were specific for the ERE1- and ERE2-containing regions of *ERα* intron 1. Input was used as a positive control. A reaction with no DNA (w/o DNA) and a reaction with b-actin-incubated chromatin for each of the ERE1 or ERE2 elements were used as negative immunoprecipitation controls.

Since CarB cells presented an over 3-fold elevation in ERα levels compared to the immortalized C5N cell line (Figure 1E), while at the same time having almost undetectable ERβ levels, they were deemed as ideal candidates for investigating the effects of selective ERα inhibition in aggressive stages of skin cancer. To this end, CarB cells were stably transfected with a previously described [16] effective dnERα mutant, S554fs, which carries a frameshift mutation at residue 554 of the ligand binding domain (Fig. 2A). Three independently obtained, stably transfected cell lines were produced, designated as C-ER 3, 4 and 7. Expression of the human dnERα in all three transfectants in comparison with control parental CarB and empty vector-transfected (CarB-V) cells was confirmed by immunocytochemistry using the only commercially available human-specific ERα antibody with documented lack of cross-reactivity with mouse ERα (code LS-B2664, LifeSpan Biosciences, WA, USA) (data not shown). In order to monitor whether S554fs expression affects endogenous mouse ERα levels, we performed Western blot analysis using an antibody specific for both human and mouse ERα (code sc-2707, Santa Cruz, CA, USA). The exogenous S554fs protein has the same molecular weight as the wild type ERα protein and, thus, the two ERα forms are indistinguishable by Western blot [17]. Therefore, mouse ERα levels were indirectly estimated by comparing the total ERα protein levels in all three clones (i.e., endogenous ERα plus exogenous S554fs) with the endogenous ERα levels of equally-loaded CarB and empty vector CarB-V cell extracts. As shown in Figure 2B, introduction of the empty vector in CarB cells does not alter endogenous ERα levels. Lack of interference of exogenous S554fs protein with the endogenous ERα levels would result in total ERα protein in all three clones, detected by anti-ERα (sc-2707), being higher than the baseline, endogenous ERα levels of CarB and CarB-V cells. Intriguingly, however, we found that the total levels of the protein product were lower than the baseline ERα levels, indicating a negative effect of dnERα protein on the endogenous ERα expression.

### dnERα Reduces ERα Binding to Conserved EREs in the Spindle CarB Cell Line

ERα binding to estrogen responsive elements (EREs) also decreased upon introduction of the S554fs-containing plasmid in CarB cells, as demonstrated by electrophoretic mobility shift assays (EMSAs) performed in protein extracts from C-ER3, C-ER4 and C-ER7 clones, using the well-characterized ERE sequence of the vitellogenin promoter (vitERE), compared to Car-B and CarB-V cells (Fig. 2C). Therefore, introduction of the dnERα mutant causes decrease of ERα protein expression along with decrease of ERα binding to ERE-containing targets in CarB cells. Since S554fs is known to exert its inhibitory effects only by binding to EREs as an inactive S554fs-ERα heterodimer [17], we examined the possibility that this negative effect of S554fs in the wild type ERα levels of C-ER 3, 4 and 7 clones is direct and could be attributed to downregulation of the *ERα* gene itself via putative ERE sequences on mouse *ERα* promoter. Using the ConTra bioinformatics tool, we searched for conserved *ERα* binding sites located in regions of mouse *ERα* promoter that show high homology among various species, including *Homo sapiens*, *Bos taurus*, *Canis familiaris*, *Equus caballus*, *Pan troglodytes*, *Pongo pygmaeus*, *Macaca mulatta* and *Rattus norvegicus*. Indeed, the *in silico* analysis revealed two highly conserved putative ERE elements both located downstream of the transcription start site (TSS), within the intron 1–2 of the 5′ UTR of the *ERα* gene (ID: ENSMUST00000105589) of *Mus musculus* (Fig. 2D). EREs span 1105 to 1123 and 1552 to 1570 bps downstream of the TSS, whereas translation of the coding region starts within exon 2, 2230 bps downstream of the TSS (Fig. 2E). ERα can bind to these EREs *in vivo*, as demonstrated by ChIP assays performed on CarB cells using an anti-ERα antibody. ERα antibody immunoprecipitated both EREs of the ΕRα promoter in CarB cells (Fig. 2F). In contrast, no PCR signal was observed when the irrelevant antibody b-actin was used for chromatin immunoprecipitation. The sheared and cross-linked DNA that was produced prior to the immunoprecipitation step (input) was used as a positive control PCR template.

### dnERα Alters the Morphology and the Oncogenic Characteristics of CarB Cells in vitro

**Figure 3:**
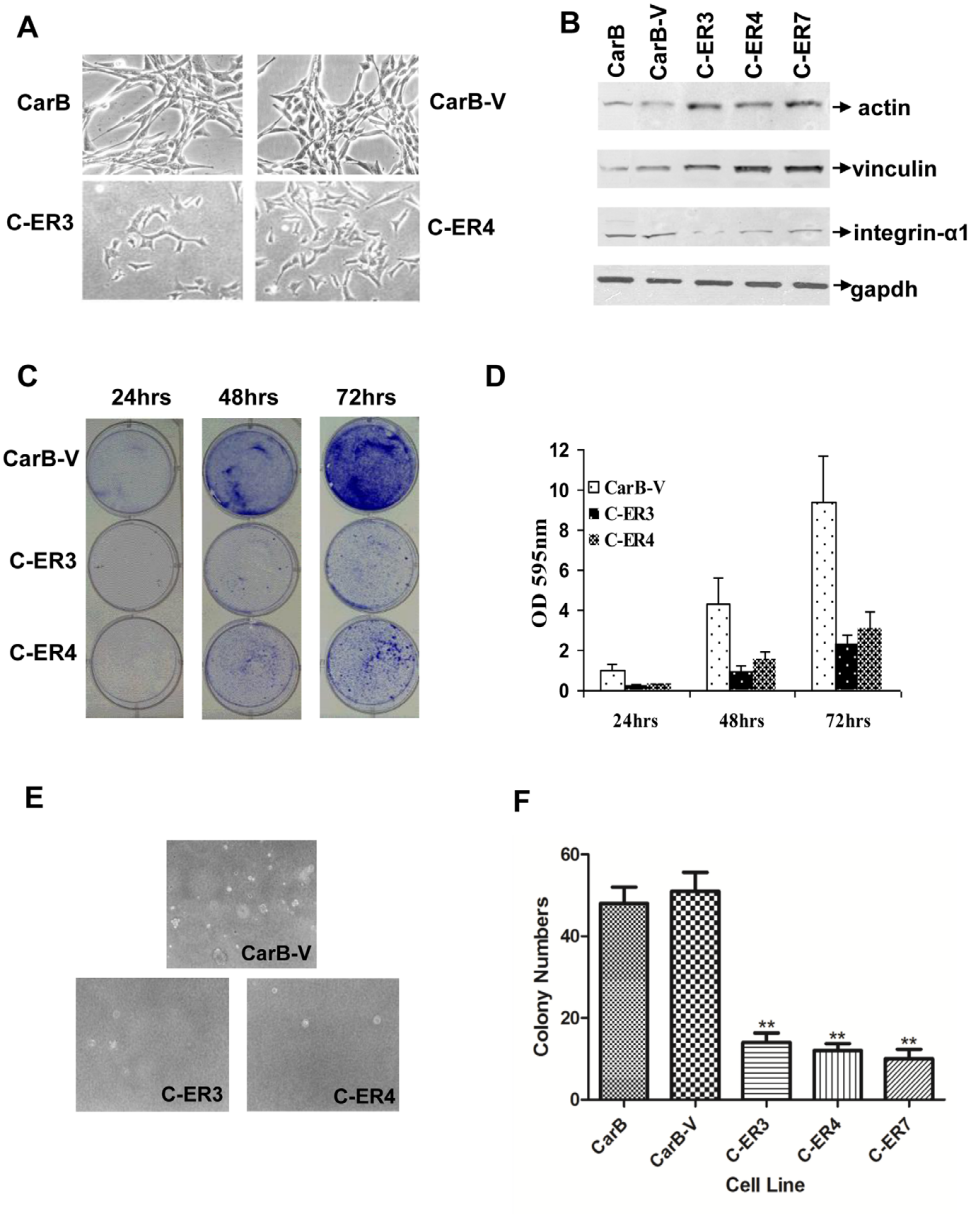
S554fs-ERα suppresses oncogenic properties of CarB-transfected cells and alters cytoskeletal and cell adhesion molecules’ levels. (A) Phenotypic characteristics of dnERα-transfected clones (C-ER3, C-ER4) in comparison with untransfected and vector-transfected CarB cells. The dnERα-transfected cells appear flattened and acquire a more epithelial morphology, whereas untransfected and vector-transfected cells present a spindle morphology. (B) Growth rate assay reveals reduction in the growth rate of C-ER3 and C-ER4 cells in comparison with control vector-transfected CarB cells. (C) Cell growing of vector-transfected CarB, C-ER3 and C-ER4 cells is expressed in OD values at 595 nm, in 24 h, 48 h and 72 h. All the results are plotted as mean ± standard deviations from three independent experiments. (D) Representative illustrations of anchorage-independent growth of CarB-V, C-ER3 and C-ER4 cells. (E) Western blot analysis for determination of levels of actin, vinculin and integrin α1 proteins in parental, CarB-V cells and S554fs-transfected CarB cells. Actin and vinculin protein expression increased, whereas integrin α1 expression decreased in all three transfected clones compared to the Car-B and CarB-V cells. GAPDH protein expression was used as a loading control. (F) Summary graph (means±SEM from triplicates) for soft agar cloning efficiency of parental control, vector-transfected and dnERα transfected spindle cell lines. Colonies were scored after 3 to 4 weeks. CarB-V is compared against CarB, C-ER3, C-ER4 and C-ER7 using t-test, and results are flagged with no asterisk when P-value is more than 0.05, with a single asterisk when the P-value is less than 0.05, with two asterisks when the P-value is less than 0.01, and three asterisks when the P-value is less than 0.001.

To further evaluate the effect of the introduction of the dnERα mutant on the tumourigenic features of CarB cell line, we monitored the morphology and growth rate of parental and S554fs-transfected cells, as well as their ability to grow on soft agar. Interestingly, we found that the S554fs-transfected CarB cells tended to lose their spindle morphology. In contrast to the characteristic spindle morphology of both parental and the CarB-V cells, dnERα-transfected cells were enlarged and flattened and became more roundish and more refractive to light (Fig. 3A). This tendency of the transfected cells to acquire a rather epithelial-like phenotype was accompanied by changes in the expression of representative cytoskeletal molecules (increased vinculin and actin levels) and extracellular matrix molecules (decreased integrin α1 levels), as shown in Fig. 3B. In addition, the S554fs-transfected cells had a slower growth rate than the parental CarB and the CarB-V cells (Fig 3C, 3D) and they finally reached confluence at day 7, while vector-transfected cells reached confluence at day 3. Soft agar assays revealed that the number of all three clones of the dnERα-transfected cell lines was dramatically reduced compared to parental or CarB-V cells (Fig. 3F), whereas their size was much smaller (Fig. 3E), revealing impaired anchorage-independent growth of dnERα transfectants. The immortalized keratinocyte cell line C5N, which produced no colonies in soft agar, was used as a negative control [18].

### dnERα Suppresses Tumour Growth in vivo

**Figure 4:**
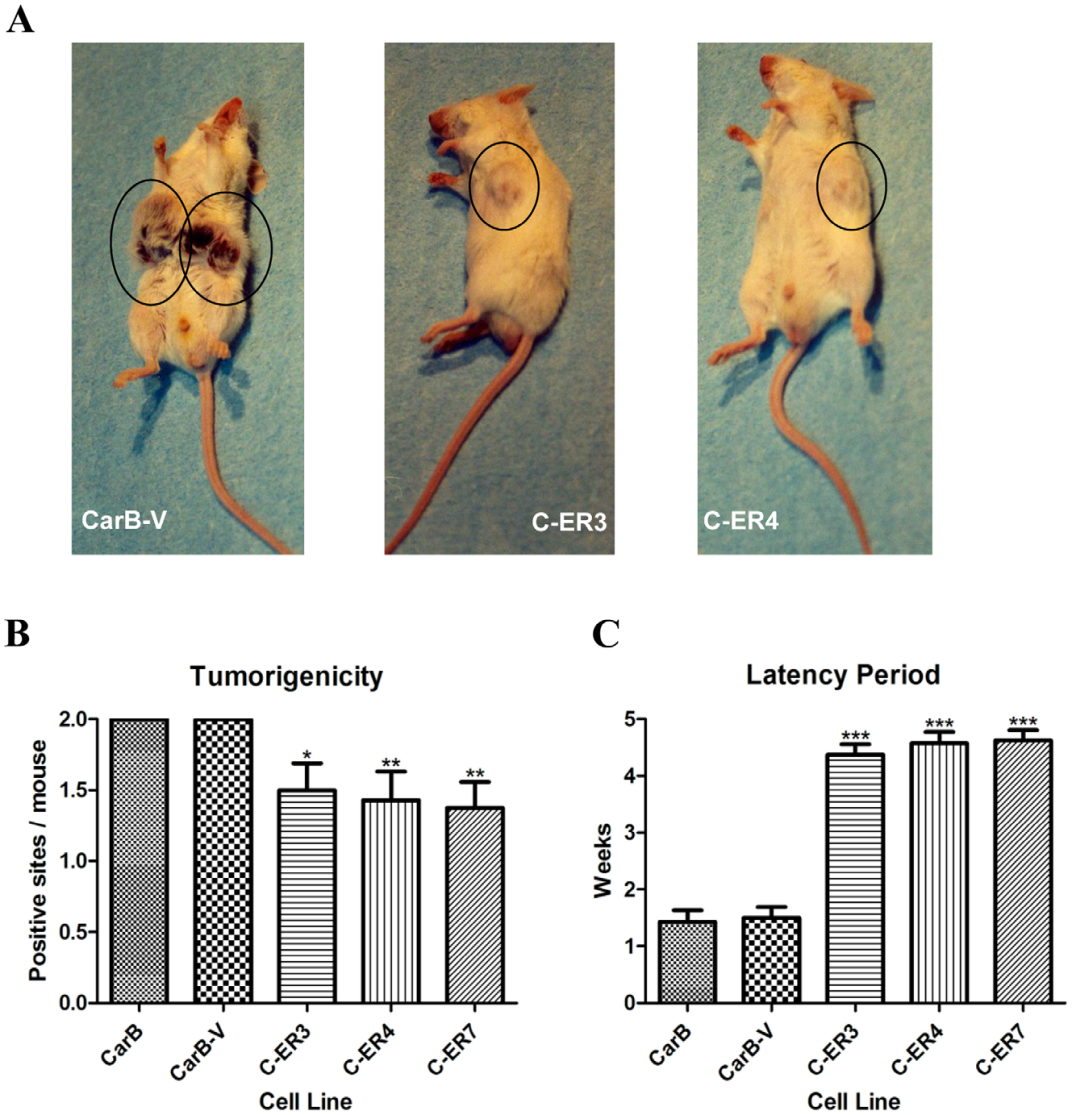
*In vivo* effect of S554fs-ERα on the biologically aggressive behaviour of mouse spindle cells. (A) Injection of C-ER3 and C-ER4 cells in BalB/c SCID mice resulted to a reduction in the number of the positive sites compared to the control CarB-V-injected mice, as well as to smaller tumours. Tumour sites are circled in black. (B) Tumorigenicity (positive sites/mouse) of control and dnERα transfected CarB cell lines. (C) Latency period (weeks) of tumours in dnERα-CarB injected mice as compared to parental and mock-transfected cells. Corresponding summary graphs (means±SEM from 7 to 8 mice) derived by comparison of mice injected with CarB-V against CarB, C-ER3, C-ER4 and C-ER7 using t-test. Results are flagged with no asterisk when P-value is more than 0.05, with a single asterisk when the P-value is less than 0.05, with two asterisks when the P-value is less than 0.01, and three asterisks when the P-value is less than 0.001.

We next assessed the *in vivo* effect of S554fs-ERα in the metastatic stages of mouse skin carcinogenesis by subcutaneously injecting dnERα-transfected cells into BALB/c SCID mice. Mice injected with the empty vector-transfected CarB or the parental CarB cells were used as controls. dnERα-CarB-injected mice developed significantly smaller tumours (approximately 0.3 cm in diameter) than the control CarB-V-injected mice (>1.5 cm in diameter) (p<0.05, t-test) (Fig. 4A). There was a reduction in the number of positive sites to the total number of injected sites of dnERα-CarB-injected mice compared to the controls (Fig. 4B). In addition, mice injected with dnERα-transfected cells present a significant prolongation of the latency period of tumour onset in comparison with the control parental or CarB-V-injected mice (Fig. 4C). Histological examination of tumours demonstrated that tumour cells from dnERα-CarB-injected mice had acquired an epithelial-like appearance and did not invade the abdominal wall, whereas tumour cells from control mice had a spindle, fibroblastoid morphology and invaded the abdominal wall (data not shown).

## Discussion

Estrogen receptors respond to estrogen hormones, particularly 17 β-estradiol (referred as E2), and directly or indirectly affect gene transcription via multiple ER-signaling pathways. In the classical ligand- and ERE-dependent signaling pathway, E2 stimulation causes ER protein molecules to dimerize and bind to specific EREs on target-gene promoters, where they recruit coactivators/regulators and trigger transcription of both nuclear and mitochondrial genes. ERE-containing genes may also respond to ER in a ligand-independent manner, possibly through stimulation of ER by intracellular kinases. Another ERE-independent signalling pathway involves interaction of E2-ER with other transcription factors, such as AP-1 and Sp1, bound to non-ERE-containing target-genes. A third non-genomic, non-transcriptional ER pathway involves a membrane-bound ER which, upon E2 stimulation, activates various protein kinases, such as MAPK, via cAMP [4], [5].

Previous studies have clearly indicated a link between ERs and skin cancer. For instance, ERβ protects against UV radiation-induced skin photocarcinogenesis probably via immunological pathways, and specifically by inhibiting photoimmune suppression, a known risk factor for cancer skin development [19]–[21]. On the other hand, an inbalanced ERα and ERβ expression is implicated in chemically induced skin carcinogenesis [14]. We show here that ERα markedly increased in aggressive mouse skin tumours *in vivo* as compared to the papilloma tumours, whereas low papilloma ERβ levels become even lower in the aggressive spindle tumours. Moreover, using a mouse skin carcinogenesis study model, we show that ERα protein levels gradually increase during the promotion and progression stages of skin cancer, peaking at the most aggressive stage, which is represented by the CarB cell line. In contrast, ERβ levels only slightly changed throughout skin carcinogenesis, being lowest in CarB cells. Consequently, the corresponding ERα/ERβ ratio in these cell lines generally increased in promotion and progression stages of mouse skin carcinogenesis in comparison with the immortalized C5N cell line, presenting the highest ratio in the most aggressive CarB cells.

The *in vitro* and *in vivo* effects of reduction of the deregulated ERα levels were next assessed by stably transfecting CarB spindle cell line with S554fs, a well-studied dnERα mutant, which has been characterized as a potent suppressor of ER-mediated transcription in breast cancer cells [22]. It has been shown that this dnERα mutant never binds to EREs as a homodimer [16] and that it causes transcriptional silencing through heterodimerization with wild type ERα monomers and antagonism with wild type ERα homodimers for ERE binding. Interestingly, we observed that introduction of this specific dominant negative (dn) mutant in CarB cells additionally resulted in a reduction of the endogenous ERα levels, along with the expected decrease of ERα-ERE complexes. This is attributed to two highly conserved, functional ERE sequences within intron 1–2 of the mouse *ERα* gene promoter, through which S554fs is plausibly able to directly exert its inhibitory effects. Therefore, activation of mouse *ERα* gene may be repressed through competition of the inactive wtERα/S554fs-ERα heterodimers with functional ERα homodimers for binding to ERE sequences on the mouse *ERα* promoter itself. This leads to inhibition of ERα synthesis and gradual reduction of the ERα molecules in the cell’s ERα reservoir that are available for composition of functional ERα homodimers, thus resulting in further potentiation of the inhibitory transcriptional effects exerted by the initially formed inactive wtERα/S554fs heterodimers. Notably, a study has demonstrated that the human *ERα* promoter contains a putative ERE which is autoregulated by ERα itself [23]. Taken together, these findings suggest that there might be a possible conserved mechanism of autoregulation of *ERα* gene and that powerful dnERα mutants such as S554fs could interfere with this mechanism to mitigate the proliferative effects of deregulated ERα expression. However, further studies are required to validate this hypothesis.

Dominant negative forms of ER have shown antiproliferative and tumour inhibitory effects upon introduction in several types of cancer cells. For instance, adenovirus-mediated delivery of S554fs mutant in the ER-positive MCF-7 breast cancer cell line effectively suppresses estrogen-stimulated cell proliferation and hormonal induction of endogenous genes [24]. Moreover, other dn ER mutants effectively induce apoptosis *in vitro* and inhibit tumour growth *in vivo* in breast cancer [25] as well as in leiomyomas [26]. In agreement with the above studies, we provide evidence on interference of dnERα with the oncogenic and aggressive characteristics of skin cancer cells, as well as with skin tumour growth. The dnERα-transfected CarB cells exhibit suppression of anchorage-independent growth, a key property of malignant cells *in vitro*, whereas they generate fewer and smaller tumours with extended latency period of tumour onset in severe combined immunodeficient (SCID) mice. Overall, the above data provide optimistic insights on the use of dn forms of ER in the targeting of ER-mediated oncogenic activities in several cancer types.

Moreover, our study provides evidence of correlation of an imbalanced ERα/ERβ ratio with the expression of molecules that affect shape and adhesion of skin cancer cells, since introduction of dnERα in the aggressive metastatic CarB cell line increases vinculin and actin protein levels and decreases integrin α1 protein levels. The effect of the dnERα mutant on cell shape and expression of cytoskeletal proteins in CarB cells presents similarities with that of the selective ER modulator, tamoxifen, in MCF-7 cells, which has been shown to increase vinculin and actin levels, promote rearrangement of cytoskeletal structures and result in cells with a more roundish and flattened morphology [27]. Alterations in molecules that influence cell shape and adhesion have been correlated with conversion of mouse skin carcinomas of epithelial morphology to spindle cell carcinomas of higher metastatic potential [28]. Therefore, it is likely that one way of suppression of the aggressiveness of CarB cell line by dnERα may involve cell structural alterations which ultimately result to reversion of the spindle phenotype.

Furthermore, integrins enable cell adhesion and transduce intracellular signals that promote cell survival and migration [29], [30] and they have recently been linked to both skin cell proliferation and ER expression. In particular, studies in integrin α1-null mice revealed that integrin α1 induces skin cell proliferation [31]. On the other hand, Lindberg *et al* reported that integrin α1 levels are affected by ERs in human breast cancer cells, possibly via a putative ERE within a region extending 10 kbps upstream of the transcription start site (TSS) of the human integrin α1 gene [32]. In concordance with these findings, we have also observed alterations in the integrin α1 levels upon introduction of dnERα in CarB cells. Moreover, we have found a putative ERE positioned −4297 to −4279 bps upstream of the TSS of the integrin α1 gene, which is highly conserved among 11 mammalian species, including humans (unpublished data). In this context, an intriguing question that should be investigated in future studies is whether the observed reduction of integrin α1 in dnERα-transfected CarB cells is mediated by ERE-dependent pathways and whether it is linked to the impaired anchorage-independent growth and/or the reduced proliferation rate that lead to the mitigation of the oncogenic features of malignant CarB cells.

Overall, our data strongly suggest that ERα-specific targeting can effectively reverse the aggressive malignant mouse skin phenotypes. This reversion is reflected in the loss of spindle morphology, which could be a result of respective changes in the levels of critical cytoskeletal and cell adhesion molecules. Still, the identification of putative, direct or indirect ER gene targets in skin carcinogenesis and the documentation of an axis between ER and cell structure/adhesion molecules that may underlie ER oncogenic effects are subjects for fruitful research. Oncoming cutting-edge, high-throughput analyses of dnERα-suppressed cells versus parental cancer cells will further assist identification of the direct and indirect targets of ERα that mediate its oncogenic effects in this cancer type.

With respect to skin cancer therapy, thorough understanding of the role of ERs and optimization of ER-targeting could ultimately enable improvement of current treatment regimes and alleviate obstacles in the management of skin cancer risk. In particular, the failure of patients with aggressive skin cancer to respond to the conventional systemic chemotherapy [1], as well as the poor tolerance of special groups of skin cancer-prone patients (e.g. immunosuppressed transplant recipients) in chemoprophylaxis [33] necessitate the development of new, alternative strategies for effective and safe molecular therapeutics against this cancer type. An example of such an approach suggested recently is tissue-specific intervention in ER-mediated oncogenesis pathways [34]. The consistent tumour growth-suppressive effects of dn forms of ERα in ERα-positive epithelial tumours [25,26 and this study], encourage experimental attempts towards the introduction of dnERα in tissue-specific targeting in the setting of the skin cancer treatment.

## Materials and Methods

### Ethics Statement

Experiments with mice were performed in the authorized animal house of the National Hellenic Research Foundation. Experiments complied with the Protocol on the Protection and Welfare of Animals, as obliged by the rules of the National Hellenic Research Foundation, the regulations of the National Bioethics Committee and the article 3 of the presidential decree 160/1991 (in line with 86/609/EEC directive) regarding the protection of experimental animals. Please note that all experiments with mice were performed according to the local, institutional and EU guidelines for the protection and welfare of animals (NHRF Bioethics Committee). These experiments are always performed in the legal and authorized in-house animal facility of our institution.

### Two-stage Chemical Carcinogenesis Protocol

The backs of 10-week old mice were shaved and treated with a single application of DMBA (25 µg in 200 µL acetone) followed by a biweekly application of TPA (200 µL of 10^−4 ^M solution in acetone) for 20 weeks. Mice were visually examined weekly and were sacrificed if moribund, if any individual tumour reached a diameter of 1 cm, or at the termination of the experiments. Tumours from sacrificed animals were snap-frozen in liquid nitrogen, fixed in formalin, embedded in paraffin and were sectioned for immunohistochemical staining.

### Immunohistochemical Staining

Immunohistochemistry was performed as described earlier [18]. The sections were stained with ERα (sc-7207; Santa Cruz, Santa Cruz, CA, USA) and ERβ (sc-8974; Santa Cruz, Santa Cruz, CA, USA) primary antibodies in a 1∶200 and 1∶100 dilution, respectively.

### Cells and Culture Conditions

Cell lines were produced and kindly provided by Dr. A. Balmain (reviewed in [3]). All cell lines were maintained in Dulbecco’s modified Eagle’s medium (DMEM) supplemented with 10% fetal bovine serum (FBS) and penicillin/streptomycin. Cells were incubated in a humidified atmosphere with 5% CO_2_ at 37°C. The ERα-ΗΕΚ and ERβ-ΗΕΚ stably transfected clones were a kind gift of Dr. M. Alexis [15].

### Preparation of Total Cell Lysates

Cells were washed twice in ice-cold phosphate-buffered saline and lysed in lysis buffer (20 mM Tris pH 7.6, 0.5% Triton X-100, 25 mM NaCl, 3 mM EDTA, 3 mM EGTA, 10 µg/mL pefabloc, 2 mM sodium orthovanadate, 10 µg/mL aprotinin, 10 µg/mL leupeptin and 1 mM dithiotreitol). Cells were incubated on ice for 30 min and centrifuged at 12,000 rpm, 4°C for 10 min. Protein estimations were performed by the Bradford method [35].

### Western Blot Analysis

Total cell lysates (40 µg) were electrophoresed on a 10% SDS-polyacrylamide gel under reducing conditions. Proteins were transferred to nitrocellulose membrane and blocked for 1 h at room temperature in 5% non-fat dry milk with TBS-0.1% Tween 20. The blots were subsequently incubated overnight at 4°C with the corresponding primary antibodies. Primary antibodies used were purchased from Santa Cruz (anti-ERα, code sc-7207; anti-ERβ, code sc-8974; anti-actin, code sc-1616; anti-integrin α1, code sc-10728; anti-vinculin, sc-25336). Anti-GAPDH (sc-20357, Santa Cruz, Santa Cruz, CA, USA) was used as a loading control. Primary antibodies were diluted 1∶1000 to 1∶2000 in 5% non-fat dry milk with TBS-0.1%-Tween 20. After three 10-min washes in TBS-0.1% Tween 20, the blots were incubated with the corresponding horseradish peroxidase-conjugated secondary antibodies (1∶5000 dilution in 1% non-fat dry milk with TBS-1% Tween 20) for 2 h at room temperature. Detection of protein levels was carried out using an enhanced chemiluminescence system (Thermo Scientific-Pierce, IL, USA). Protein levels were normalized to GAPDH and quantification was performed as previously described [36].

### Electrophoretic Mobility Shift Assay

Annealed oligonucleotides for the vitellogenin Estrogen Responsive Element (5′-AGC TTC AAA GTC AGG TCA CAG TGA CCT GAT CAA AGA-3′) and (5′-AGC TTC TTT GAT CAG GTC ACT GTG ACC TGA CTT TGA-3′) [37] were end-labelled with γ^32^P-ATP using T4 polynucleotide kinase and the reaction products were purified on a 8% polyacrylamide gel. DNA binding reactions were carried out by mixing 2,000 cpm of [γ-^32^P]ATP-labeled oligonucleotide with 20 µg of total cell protein in binding buffer [50 mM HEPES (pH 8.0), 500 mM NaCl, 0.5 M PMSF, 0.5 mg/mL BSA, 20% glycerol, 1 mM EDTA) plus 1 mM DTT and 150 µg/mL poly(dI-dC) (Sigma-Aldrich, St Louis, MO, USA). The reaction mixture was left at room temperature for 30 min and the samples were subsequently subjected to electrophoresis on a 6% polyacrylamide gel at 150 V for 90 min, dried and visualized by autoradiography.

### Chip Assay

Chromatin immunoprecipitation assays in CarB cells were performed as previously described [36]. Chromatin was precipitated either with ERα antibody (kindly provided by Dr. M. Alexis) [15] or b-actin antibody (Santa Cruz, CA, USA). The pelleted DNA was resuspended in 10 µL and amplified by PCR. The following PCR primers were used: for ERE1, forward: CGCCAGAGCTTTAGTCAAGG, reverse: CACACTTGGGTGTCCCTACC; for ERE2 forward: ACACACCCCATCCTATTTGC, reverse: GCCAAGCTCCAATCTGTCTC.

### Plasmids and Transfections

CarB cells were transfected with a mixture of a plasmid containing the S554fs dominant negative ERα frameshift mutant [16] (kindly provided by Dr. B. Katzenellenbogen) and a pCMVneo plasmid (which confers resistance to geneticin) in a 10∶1 ratio, using the calcium phosphate method [38]. Control CarB-V cells were transfected by pCMVneo alone. Following 18 h of incubation, the cells were washed twice in PBS and incubated for 24 h in fresh DMEM medium supplemented with 10% FBS. Twenty four hours later the cells were diluted and incubated in DMEM supplemented with 10% FBS under selective conditions (1000 µg/mL geneticin) (Sigma-Aldrich, St Louis, MO, USA) for 2 weeks. Individual geneticin-resistant colonies were isolated with cylinder trypsinization and were grown in DMEM supplemented with 10% FBS.

### Growth Rate Assay

4×10^4^ cells were plated in 6-well culture dishes and allowed to grow in DMEM supplemented with 10% FBS. The growth rates of parental and transfected cell lines were compared 24, 48 and 72 h after plating of cells. The cells were methanol-fixed and stained with 0.5% crystal violet solution. Excess dye was removed under running tap water and the dishes were air dried and destained with 33% acetic acid. The absorbance was measured at 595 nm. The assay was performed in triplicate.

### Anchorage-independent Growth

5×10^3^ cells were plated onto 3 mL DMEM containing 10% FBS and 0.3% agar, over a solidified cushion of 2 mL DMEM containing 10% FBS and 0.6% agar. Cells were allowed to grow for two weeks and 0.5 mL of fresh DMEM supplemented with 10% FBS was added into the wells every 2 days. Individual macroscopic colonies were finally counted [39]. Colonies were scored after 3 to 4 weeks and each value was calculated as an averaged score of three independent experiments.

### In vivo Tumourigenicity Studies

Parental CarB, control vector-transfected CarB-V and S554fs stable transfectant cells, were harvested, washed and resuspended in PBS. Approximately 10^6^ cells were injected subcutaneously at two sites in the abdominal region of female 6-week-old Balb/c severe combined immunodeficient (SCID) mice (The Jackson Laboratory, Bar Harbor, ME). This strain is routinely used in oncogenicity protocols to avoid bias due to immunological responses of mice to injected cells. Tumour growth was monitored 2 times weekly and animals were killed when tumours reached a diameter or approximately 1.5 cm or at the end of the observation period.

### Bioinformatic Analysis

GeneCards [40] was used for the identification of the HGNC gene symbols of the genes of interest. ConTra [41], a promoter alignment analysis web tool for identification of EREs across species was used, as follows. For each gene symbol, the Ensembl [42] transcript with most downstream Transcription Start Site (TSS) was selected. The sequences were compared against V$ER_Q6 TRANSFAC position weight matrix of ERα target motifs using MATCH algorithm [43] with a core cutoff of 0.95 and a similarity matrix cut-off of 0.85. ConTra output genomic sequence alignments where potential ER binding sites appeared in colour. Conserved potential ER binding sites were identified, through visual inspection.
